# Role of miRNAs in Melanoma Metastasis

**DOI:** 10.3390/cancers11030326

**Published:** 2019-03-07

**Authors:** Anna Gajos-Michniewicz, Malgorzata Czyz

**Affiliations:** Department of Molecular Biology of Cancer, Medical University of Lodz, 6/8 Mazowiecka Street, 92-215 Lodz, Poland; anna.gajos-michniewicz@umed.lodz.pl

**Keywords:** miRNA, exosomes, melanoma, metastasis, drug resistance, pre-metastatic niche, invasion

## Abstract

Tumour metastasis is a multistep process. Melanoma is a highly aggressive cancer and metastasis accounts for the majority of patient deaths. microRNAs (miRNAs) are non-coding RNAs that affect the expression of their target genes. When aberrantly expressed they contribute to the development of melanoma. While miRNAs can act locally in the cell where they are synthesized, they can also influence the phenotype of neighboring melanoma cells or execute their function in the direct tumour microenvironment by modulating ECM (extracellular matrix) and the activity of fibroblasts, endothelial cells, and immune cells. miRNAs are involved in all stages of melanoma metastasis, including intravasation into the lumina of vessels, survival during circulation in cardiovascular or lymphatic systems, extravasation, and formation of the pre-metastatic niche in distant organs. miRNAs contribute to metabolic alterations that provide a selective advantage during melanoma progression. They play an important role in the development of drug resistance, including resistance to targeted therapies and immunotherapies. Distinct profiles of miRNA expression are detected at each step of melanoma development. Since miRNAs can be detected in liquid biopsies, they are considered biomarkers of early disease stages or response to treatment. This review summarizes recent findings regarding the role of miRNAs in melanoma metastasis.

## 1. Introduction

Melanoma develops from the malignant transformation of neural crest-derived melanocytes. Primary melanoma can be easily treated by surgical resection. Invasive melanoma cells escape from their primary location and translocate via the circulatory system or body cavities to lymph nodes or distant organs to establish a secondary cancer tissue. Advanced melanoma is promoted by a complex interplay between melanoma cells and components of the microenvironment [1,2]. Melanoma in unresectable advanced stages has a very high frequency of acquired mutations that are critically involved in proliferation, survival, and metastasis [2,3]. The discovery of frequent functional mutations has led to new molecular classifications of melanoma and created the opportunity to develop specific inhibitors. Approximately 50% of melanomas harbor mutations in *BRAF* (V-raf murine sarcoma viral oncogene homolog B1), leading to the aberrant activation of the MAPK signaling pathway [4]. Vemurafenib and dabrafenib were the first and second FDA (Food and Drug Administration) approved targeted therapies for BRAF-mutated melanomas, respectively, and then combination therapies of BRAF and MEK inhibitors became available [5]. Unfortunately, the majority of melanomas are either intrinsically resistant or develop resistance within a few months after initial treatment [6,7]. In addition to targeted therapies that employ small molecules, immunotherapies have been developed, where immune checkpoint blockers (ipilimumab, nivolumab, pembrolizumab) re-activate cytotoxic T cells to eliminate melanoma cells [8,9]. Although a prolonged clinical benefit from immunotherapies especially combining anti-PD-1 and anti-CTLA-4 is evident, predictive biomarkers that can unambiguously identify responders are still unavailable in clinical practice [10]. Between 2011 and 2016, ten new targeted and immune therapies were approved for the treatment of metastatic melanomas [11]. A new algorithm for treating patients with metastatic melanoma has been proposed to maximize therapeutic benefit while limiting toxicity [12]. Melanoma heterogeneity—featured as diverse genomes, transcriptomes, epigenomes, and proteomes within a tumour—and cellular plasticity, which does not involve mutations, are the major obstacles that limit durable therapeutic responses [13]. miRNAs are epigenetic factors that control a plethora of processes including cell proliferation and differentiation, cell senescence, survival, autophagy, and migration, and contribute to changes in cellular metabolism and genome stability. Therefore, even slight alterations in miRNA levels can result in various pathologies, including cancer (for review: [14,15,16,17,18]). OncomiRs are miRNAs associated with carcinogenesis, which affect the following hallmarks of cancer: (1) self-sufficiency in growth signals, (2) insensitivity to anti-growth signals, (3) evasion from apoptosis, (4) limitless replicative potential, (5) angiogenesis, (6) invasion, (7) metastasis, and (8) tumour-promoting inflammation [19,20]. miRNAs are recognized as important contributors to melanoma biology, and dysregulated miRNA expression is associated with melanoma progression [21,22,23,24,25,26]. Aberrant expression of miRNAs in melanoma cells compared to melanocytes [20,26,27,28] is a result of chromosomal abnormalities, epigenetic regulation, and other disturbances of miRNA biogenesis [23,29]. It has been demonstrated that the MAPK signaling pathway, which is upregulated in melanoma, controls a network of 420 miRNAs [30]. In addition, miRNA dysregulation has been observed during different stages of melanoma, and miRNAs are considered as biomarkers with potential diagnostic and prognostic usefulness [21,22,23,31,32]. The complex contribution of miRNAs is further reflected by their participation in forming the tumour niche, not only at the primary tumour site, but also in distant tissues [33,34]. This review focuses on the influence of miRNAs on processes that dictate melanoma progression and metastasis.

## 2. Biogenesis, Function, and Extracellular Transport of miRNAs

**Figure 1:**
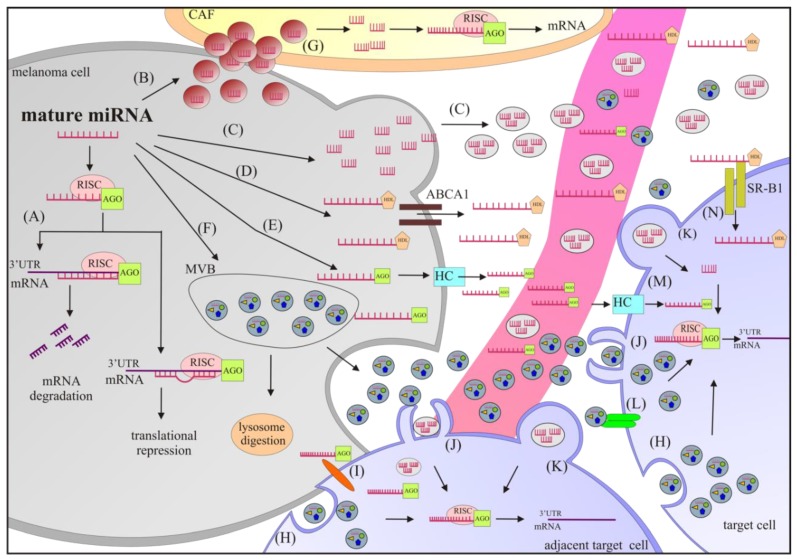
A schematic representation of the functions and cellular release/uptake of miRNAs. (**A**) In the cytosol, mature miRNAs associated with the protein AGO (argonaute) are incorporated into the RNA-induced silencing complex (RISC) that binds to target mRNAs to induce degradation (perfect binding) or repress translation (imperfect binding). In addition, mature miRNAs can be exported out of the cell and transported to recipient cells by various carriers, including melanosomes (B), ectosomes (C), HDL (high density lipoprotein) (D), AGO (E), and exosomes (F). To disseminate to distant cells, miRNAs must enter the cardiovascular or lymphatic system. Mechanisms of miRNA uptake by the target cell include direct melanosome uptake (G), endocytosis (H), gap junctions (I), phagocytosis (J), fusion (K), and receptor-mediated uptake (L). Target cells include melanoma cells, lung cells, endothelial cells, and bone marrow-derived cells. ABCA1: ATP-binding cassette transporter; CAF: cancer associated fibroblast; HC: hemichannel; MVB: multivesicular body; and SR-B1: scavenger receptor class B type 1.

miRNAs are a group of small (19–25 nt), endogenously expressed, non-protein-coding RNAs that regulate gene expression at the post-transcriptional level. Since the discovery of miRNAs in 1993 by Lee and colleagues in the nematode *Caenorhabditis elegans* [35], the number of new miRNAs has been increasing each year, and according to the miRBase database, 38,589 hairpin precursor miRNAs producing 48,885 mature miRNAs in 271 species have been registered so far (release 22.1, http://www.mirbase.org). The biogenesis of miRNAs and their dysregulation in cancer have been comprehensively described [14,23,36,37,38,39]. The primary function of miRNA is to bind to a target sequence in the form of an mRNA in order to interfere with the translation machinery, thereby altering or preventing protein synthesis [36,40]. Perfect or near-perfect complementarity beyond the seed region sequence results in mRNA degradation, whereas imperfect binding results in translational attenuation [40,41] (Figure 1). The function of RNA–miRNA interactions, beyond repression of gene expression, has been recently demonstrated [41,42,43]. A large number of non-canonical binding sites for miRNAs have been identified in mRNAs, and several mRNAs are considered sponges that sequester tumour-suppressive miRNAs [44]. These aberrant miRNA–RNA interactions can contribute to the development of melanoma [45] and may be preserved in drug-resistant melanoma cells [46].

miRNAs are crucial messengers of intercellular communication. The extracellular transfer of miRNAs is mediated by either protein transporters such as AGO (argonaute), HDL (high density lipoprotein), or extracellular vesicles (EV), e.g., oncosomes, ectosomes, exosomes, and melanosomes [33,47,48,49,50,51,52]. miRNA aggregation with proteins or encapsulation into EVs protect them from degradation by RNases [34,53,54,55]. Oncosomes (1–10 μm), first described in 2008 [56], are tumour-derived microvesicles that originate from membrane shedding of cancer cells, and their amount is associated with tumour aggressiveness and progression [50,52]. Ectosomes (50–2000 nm) are formed through the outward budding of the plasma membrane [53]. Exosomes, which are the smallest type of EVs (30–180 nm in diameter), were discovered by Pan and Johnstone in 1983 [57], and originate from intracellular endosomes that subsequently form multivesicular bodies (MVBs) that are released from donor cells upon fusion with the plasma membrane [58,59]. Alternatively, MVBs can be degraded in lysosomes [60]. Originally, exosomes were thought to play a major role in cellular debris disposal, but nowadays they are recognized as important transporters of various important bioactive molecules such as miRNAs, tRNAs, mRNAs, lncRNAs, piRNAs, rRNAs, snRNA, snoRNAs, DNA fragments, lipids and proteins, and they are considered predictive and prognostic biomarkers useful in monitoring tumour development and results of treatment [10,61]. According to the Exocarta database, which gathers information about exosomal contents, 9769 proteins, 3408 mRNAs, 2838 miRNAs, and 1116 lipids have been identified in exosomes from different cell types of multiple organisms (http://www.exocarta.org). Exosomal miRNAs substantially contribute to melanoma progression by transferring information between melanoma cells themselves, as well as melanoma cells and neighboring non-tumour cells such as fibroblasts and endothelial cells [62,63]. miRNAs modulate the properties of immune cells and cells at distant niches [62,63]. Intercellular miRNA transport mediated via EVs includes: fusion, endocytosis, phagocytosis, and receptor mediated uptake. AGO-bound miRNAs have been shown to pass between adjacent cells via gap junctions or through hemichannels (HC) into the extracellular space to enter the target cell [53]. Transporters such as ABCA1 (ATP-binding cassette transporter 1) mediate the release of HDL–miRNA complexes that are taken up by recipient cells via SR-B1 (scavenger receptor class B type 1) [64]. A schematic representation of miRNA function and transport is summarized in Figure 1.

## 3. miRNAs Modulate the Tumour Niche within the Dermis

Normal melanocytes produce melanin, which is transferred in melanosomes to keratinocytes to induce skin pigmentation. Melanoma cells retain the ability to transfer melanosomes to neighboring cells, which contributes to the formation of the tumour niche within the dermis [33,65]. Dror et al. have shown that melanoma cells communicate with fibroblasts via melanosomes that are released into the dermis prior to melanoma cell invasion [33]. Moreover, they have demonstrated melanosomal transport of miR-211, which contributes to the reprogramming of primary fibroblasts into cancer-associated fibroblasts (CAFs). CAFs play an important role in the progression from primary to metastatic melanoma. Increased proliferation of CAFs has been shown to be associated with a reduced level of IGF2R (insulin growth factor 2 receptor), and IGF2R mRNA was found to be a direct target of miR-211 [33]. Moreover, miR-211 transfected into fibroblasts upregulates phospho-ERK, which indicates activation of the MAPK signaling pathway. The spectrum of miRNAs transferred by melanoma-derived melanosomes is different from melanosomes secreted by melanocytes, and lower miR-211 levels have been observed in the dermis of normal skin compared with melanoma in situ. These findings suggest that melanoma cells are capable of modulating the stromal niche during the early phase of the disease by altering the phenotype of dermal fibroblasts. Thus, blocking the release of melanoma-derived melanosomes might forestall early dermal changes [33].

Another mutation-independent mechanism that underlies the initiation of melanoma metastasis during the transition from radial to vertical growth involves miR-222/221, NOTCH, and MITF [66]. In melanoma cells that are present in a NOTCH ligand-free microenvironment, the transcription factor MITF represses the *miR-222/221* promoter. During radial growth, which occurs when melanoma cells establish contact with distal differentiated keratinocytes that express NOTCH ligands, the activated intracellular domain of NOTCH interferes with MITF binding to the *miR-222/221* promoter. The increased expression of miR-222/221 therefore enables the acquisition of invasion capabilities [66]. It has been shown that the level of oncomiR-222/221 inversely correlates with the level of tumour suppressor miR-126/miR-126* during melanoma progression [67]. Felli et al. have reported high expression of miR-126/126* in melanocytes and primary vertical growth phase melanomas, with its decrease in subcutaneous and lymph-node metastases, whereas *miR-221/222* expression has been almost undetectable in melanocytes and gradually increased during melanoma progression [67]. Protein analyses have revealed the reverse expression pattern of several factors, targeted by miR-126/126* and induced by miR-222/221, or the opposite. Interestingly, the dual regulation of AP2α (activating protein 2 alpha), a transcription factor that is lost during transition from primary to local and metastatic dissemination, has been revealed. While AP2α plays a role of a transcriptional activator of tumour suppressor miR-126/126*, AP2α transcripts are targeted by oncogenic miR-222/221 [67]. Alterations in this auto-sustaining loop during melanoma development may contribute to changes in the expression of several pro-oncogenic factors, including cell adhesion molecules, extracellular matrix (ECM) degrading enzymes, angiogenic and survival proteins. Increased expression of *miR-221/222* in melanoma cell lines has been associated with induced proliferation, migration, and invasion of melanoma cells in vitro and in vivo compared to normal melanocytes [68]. The expression of *miRNA-221/222* is transcriptionally downregulated by PLZF (promyelocytic leukaemia zinc finger) or antagomir-221/222 treatment [68]. miR-222 is part of the melanoma exosomal cargo, and exosomes released by miR-222-overexpressing cells are taken up by recipient primary melanoma cells to promote tumour growth through the activation of the PI3K/AKT pathway, and downregulation of the tumour suppressor p27 [69]. Transcripts of c-KIT and the cyclin-dependent kinase inhibitor p27 are targets of miR-221/222. miR-221-induced attenuation of p27 and c-KIT has been shown in another study [70]. Inactivating point mutations in p27 are rare, and p27 is mostly regulated at the posttranscriptional and posttranslational levels. Thus, a proposed miR-221/222-based mechanism that blocks p27 translation represents an additional oncogenic program that triggers an abnormal cell cycle rate in melanoma cells [68].

## 4. miRNAs Modulate Angiogenesis and Immune Response, Intravasation, and Extravasation of Melanoma Cells

To disseminate into lymph nodes or distant sites, melanoma cells must enter the cardiovascular or lymphatic systems. Forming new vessels via angiogenesis, intravasation, and extravasation is important during melanoma progression. miR-203 has been shown to suppress melanoma cell migration and angiogenesis in vitro, and to decrease primary tumour growth and suppress metastasis to the lungs and lymph nodes in vivo [71]. *miR-203* expression is downregulated in metastatic melanoma compared to primary melanoma, and hypermethylation of the *miR-203* promoter has been suggested as the potential mechanism. Transcripts of SLUG (Snail Family Transcriptional Repressor 2), which contains an evolutionally conserved 8-mer-binding site in its 3′UTR, has been identified as a direct miR-203 target, and a higher level of SLUG protein has been observed in malignant melanoma samples compared with benign nevi [71]. miR-203-mediated reduction in IL-8 levels plays an important role in the modulation of neovascularization [71,72]. Pencheva et al. have revealed the involvement of miR-1908, miR-199a-5p, and miR-199a-3p in angiogenesis during melanoma progression [73]. Melanoma cells exhibiting high miR-1908, miR-199a-5p, and miR-199a-3p levels possess a greater capacity to recruit endothelial cells in vitro and in vivo [73]. Melanoma cell-derived exosomes promote the migration of endothelial cells and affect angiogenesis by transferring miR-9 to endothelial cells and activating the JAK-STAT pathway [74]. The JAK-STAT pathway is one of the major oncogenic signaling pathways in human malignancies [74]. It has been shown that miR-214 induces *MET* expression [75], which is associated with the increased expression of *VEGF-C* (vascular endothelial growth factor C) and *PDGF-C* (platelet-derived growth factor C), and enhanced lymphangiogenesis. VEGF-C induces proliferation of lymphatic endothelial cells (*LECs*), whereas PDGF-C is a mitogenic factor and chemoattractant for CAFs and blood endothelial cells [76,77,78].

The involvement of miR-214 in extravasation has been determined both in vivo and in vitro [75]. In vitro, trans-endothelial migration of miR-214 was typical for miR-214-overexpressing cells in comparison to the A375P cell line exhibiting low *miR-214* expression. In vivo, extravasation of miR-214-overexpressing cells from blood vessels stimulated the formation of lung metastasis. In contrast, decreased extravasation was characteristic of miR-214-silenced melanoma cells. Moreover, miR-214 has been shown to reduce anoikis, which could be important for melanoma survival in the blood circulation [75].

Melanoma is one of the most immunogenic tumours, and the relationship between melanoma cells and the immune system is under continuous investigation. During melanoma development, cell proliferation and survival are associated with immune editing that is a dynamic process that includes immunosurveillance, equilibrium between melanoma and immune cells, and finally evasion of the immune response [79].

Growing evidence indicates the presence of close interactions between tumour-derived miRNAs and cells of the immune system (for review: [80,81,82]). Several miRNAs were found to be involved in the regulation immune responses in melanoma. It has been demonstrated that in sentinel nodes, which represent the first immunological site where the anti-melanoma immune response becomes dysfunctional, several miRNAs are differentially expressed between progressing patients and non-progressing patients [83]. Analysis of three miRNAs, miR-30c, miR-23a, and miR-4299 suggested that miRNA involvement associated with immune suppression is cell type-specific. miR-30c, miR-23a, and miR-4299 have been found to selectively modulate the expression of CD30 in Tregs (regulatory T cells) and MDSCs (myeloid-derived suppressor cells), two key players in tumour-induced immunosuppression. Overexpression of the miR-30d/30b cluster has been shown to enhance the invasive capacity of melanoma cells in vitro, and increase their metastatic potential in vivo [84]. GALNT7 (polypeptide N-acetylgalactosaminyltransferase 7) mRNA is among the known miR-30d/30b targets. GALNT7 significantly affects the O-glycosylation of membrane proteins by interacting with the ECM in the tumour microenvironment [84]. Moreover, during melanoma progression, the upregulation of *miR-30d* expression together with the reduced expression of *GALNT7* enhances the secretion of IL-10 (interleukin 10) and decreases immune cell activation and recruitment. Furthermore, they have observed that overexpression of *miR-30d* is associated with reduced CD3^+^ T cells recruitment and induction of regulatory T cells. This can be partially associated with increased IL-10 secretion, thus the miR-30b/30d-GALNT7-IL-10 axis could be an explanation of the immunosuppressive behavior of melanoma. This is further supported by the observation that upregulated miR-30d increased the level of FOXP3 (forkhead box P3)-positive lymphocytes [84]. Natural killer (NK) cells activating receptor NKG2D (natural killer group 2, member D) and NKG2D ligands (NKG2DLs) play an important role in cancer immune surveillance [85]. Heinemann et al. have shown that ULBP2 (UL16 binding protein 2) mRNA, a surface molecule belonging to the NKG2DL group, is a target of miR-34a/miR-34c [86]. Metastatic melanoma cells escape immune surveillance by enhancing ULBP2 shedding, which makes soluble ligands detectable in serum from melanoma patients [87].

## 5. miRNAs Modulate Melanoma Cell Invasion

MITF-M, a lineage-restricted transcription factor that operates in normal melanocytes, plays an important role as a melanoma addiction oncogene [88]. According to the rheostat model [89,90], different programs can be executed in melanoma cells depending on MITF activity, and low levels and activity of MITF are connected with an invasive phenotype. MITF activity is tightly regulated at the transcriptional, post-transcriptional, and post-translational levels [91], and miRNAs including miR-137, miR-148, miR-182, miR-211, and miR-340 are involved in this regulation. Bemis et al. have shown that miR-137 downregulates *MITF* expression in melanoma cell lines [92]. *miR-137* expression has been shown to correlate with poor survival in stage IV melanoma patients [93]. Multiple oncogenic target mRNAs, including c-MET (mesenchymal to epithelial transition), YB1 (Y box-binding protein 1), EZH2 (enhancer of zeste homolog 2), and PIK3R3 (phosphatidylinositol 3 kinase regulatory 3) are downregulated by miR-137 [94]. miR-148 has been reported to negatively regulate *MITF* expression in melanoma cells through a conserved binding site in the 3′UTR sequence [95]. Combined overexpression of both miR-137 and miR-148 does not, however, result in a cumulative effect [95]. Segura et al. have found that *miR-182* is frequently amplified and upregulated in melanoma cell lines and tissue samples, and its overexpression promotes the migration and survival of melanoma cells through the direct downregulation of *MITF-M* and *FOXO3* (Forkhead box O3) expression [96]. Moreover, the expression of *miR-182* gradually increases from primary to metastatic melanomas [96]. The expression of *miR-182* is epigenetically regulated [97], and this epigenetic modification may be of clinical significance because *miR-182* is methylated in melanoma cells in a cancer-specific manner [97]. Overexpression of *miR-182* in A375 melanoma cells increases proliferation, stimulates migration and invasion, inhibits cell apoptosis, and blocks the cell cycle at the S phase [97]. Another miRNA that influences MITF levels indirectly is miR-211. The expression of *miR-211*, which resides in the sixth intron of *TRPM1*, is downregulated in primary melanomas compared with benign melanocytic nevi and melanocytes [28,98]. Furthermore, lower miR-211 levels are observed in highly invasive melanoma cell lines in comparison to less invasive cell lines [26]. miR-211 inhibits melanoma migration and invasion [99,100,101], and the loss of cell adhesion via direct regulation of NUAK1 mRNA [102]. A switch model has been proposed in which MITF and miR-211 can activate an opposite cellular program than the program driven by PAX3 and BRN2 [101,102,103]. miR-211 targets POU3F2/BRN2 (POU-domain class 3 transcription factor 2/brain-specific homeobox 2) mRNA, which is a known repressor of *MITF* expression. The loss of miR-211 results in elevated BRN2 levels and leads to the inhibition of *MITF* expression and maintenance of melanoma cells in a dedifferentiated, pro-invasive state [101]. In BRAF-mutant melanoma cells, BRN2 transcriptionally represses the expression of *PDE5A* (cyclic nucleotide phosphodiesterase 5A), leading to increased cGMP levels and phosphorylation of MYL2 (myosin light chain 2) and consequently increased invasion [104]. Furthermore, miR-211 modulates the transcript levels of IGF2R, TGFBR2 (transforming growth factor beta receptor II), and NFAT5 (nuclear factor of activated T-cells 5) [99]. miR-340 drives a decrease in *MITF* expression, which interacts with two target sites on the short 3′UTR of the MITF transcript [105]. This regulation is independent of alternative cleavage and polyadenylation, and it has been shown that CRD-BP (coding region determinant-binding protein) restricts the activity of miR-340 by preventing its access to MITF mRNA [105]. All the above data indicate that the expression of MITF, an important melanocyte- and melanoma-specific transcription factor, is under the control of several miRNAs that are either upregulated or downregulated in melanoma (Table 1).

The loss of let-7b increased the expression of basigin (BSG, CD147) and enhanced MMP (matrix metalloproteinase) production, whereas its overexpression suppressed proliferation, invasion, migration, and colony formation in the B16-F10 melanoma cell line and reduced the number of tumour nodules found in the lungs of mice intravenously injected with let-7b-transfected B16-F10 cells [119]. Changes in the level of let-7a, which is elevated in melanocytes and reduced or lost in melanoma, influence ECM (extracellular matrix) remodeling. Müller at al. have shown that let-7a is a negative regulator of *ITGB3* expression, which leads to diminished levels of integrin β3 [118]. Integrin β3 plays an important role in ECM remodeling, and its expression increases in melanoma cells [127]. Transcripts of integrin α3 and transcription factor AP-2γ (activating protein 2 gamma) are directly repressed by miR-214 binding to their 3′UTRs [75]. Several AP2-repressed genes (e.g., *MCAM-MUC18* and *VEGFA*) are upregulated in the presence of miR-214, whereas the expression of AP2-activated genes (e.g., *TGFB*, *IGFBP5* (insulin-like growth factor-binding protein 5) or *ERBB2* (ERB-B2 receptor tyrosine kinase 2)) decreases when miR-214 is overexpressed. Moreover, other AP2-regulated genes, including *CDH1* encoding E-cadherin and *TIMP1* or *TIMP2* encoding metallopeptidase inhibitors, are upregulated in melanoma cells after transfection with miR-214 precursors [75].

It has been shown that the upregulation of miR-21 induces an invasive phenotype in non-metastasizing WM1552c melanoma cells. However, its overexpression does not increase proliferation and migration of melanoma cells [108]. TIMP3 (tissue inhibitor of metalloproteinases 3) mRNA has been recognized as one of the potential targets of miR-21 [108].

Transcription factor E2F1 induces the expression of *GABRE* (epsilon subunit of gamma-aminobutyric acid A receptor), and the *miR-224/miR-452* cluster located within the intron of *GABRE* [128] is involved in an EMT (epithelial–mesenchymal transition)-like process in melanoma cells. Knoll et al. have found that miR-224/miR-452 target the metastatic suppressor TXNIP (thioredoxin-interacting protein) that induces feedback inhibition of E2F1 [116]. TXNIP has been previously implicated in increased p16 and reduced RB (retinoblastoma) phosphorylation [129]. Furthermore, ectopic expression of this miRNA cluster in less aggressive cells results in cytoskeletal rearrangement and promotes migration and invasion. Therefore, the activity of the E2F1-miR-224/452-*TXNIP* mRNA axis has been suggested to be a molecular signature that predicts patient survival [116].

Members of the miR-200 cluster (miR-200a, miR-200b, miR-200c, and miR-141) also play an important role in tumour metastasis [130,131,132]. miR-200c directly targets mRNA of *BMI-1 (B-cell-specific Moloney murine leukemia virus integration site 1)* [133], leading to increased E-cadherin expression with concomitant inhibition of cell invasion [134,135]. The loss of miR-200c is accompanied by increased *BMI1* expression and followed by upregulation of ABC transporters, as well as the activation of the PI3K/AKT and MAPK signaling pathways [122].

*miR-125b* has been shown to be overexpressed in metastatic melanomas in comparison to primary melanomas, and miR-125b inhibition decreases the invasive potential of aggressive melanoma cells in vitro [113]. The transcription of *miR-125b* is induced by TCF4 (transcription factor 4) [133], and one of the direct targets of miR-125b is a transcript of NEDD9 (neural precursor cell expressed developmentally down-regulated protein 9) [113], which is involved in the mesenchymal movement of melanoma cells [136].

miR-34b, miR-34c, and miR-199a-3p are implicated in melanoma metastasis by targeting MET mRNA [120]. Downregulation of *miR-34b*, *miR-34c,* and *miR-199a-3p* expression results in increased *MET* expression and cell migration. Liu et al. have found that *miR-675* expression is downregulated in melanoma cell lines and tissues, and its upregulation impairs the proliferation and invasiveness of these cells [126]. miR-675 targets the transcript of metadherin (MTDH) in melanoma, and the level of MTDH, which is involved in increased invasiveness of tumour cells [137], is inversely correlated with *miR-675* expression [126]. *miR-375* is epigenetically regulated in melanoma by methylation of CpG islands [138], and its ectopic expression in the WM1552C melanoma cell line has been shown to inhibit invasion [138]. *miR-375* methylation is stage-specific because it increases with the transition from normal skin to melanoma, which suggests its possible use as a diagnostic [138].

*miR-638* is overexpressed in metastatic melanomas compared with primary melanomas [117]. *miR-638* is suppressed by AP-2α, and epigenetic silencing of AP-2α-binding sequences at the *miR-638* promoter leads to the aberrant expression of *miR-638* during melanoma progression [117]. Moreover, miR-638 enhances lung colonization in vivo, as lung sections of mice, which had been injected with miR-638-transduced cells displayed more metastatic nodules than lungs of control mice. TP53INP2 (tumour protein p53 inducible nuclear protein 2) has been found to be a direct target of miR-638 [117]. miR-1908, miR-199a-5p, and miR-199a-3p are also recognized as factors promoting invasion, angiogenesis, and colonization [73]. Transcripts of apolipoprotein E (ApoE) and the heat-shock factor DNAJA4 (DnaJ Heat Shock Protein Family (Hsp40) Member A4) are direct targets of those miRNAs [73]. Interestingly, it has been found that locked nucleic acids (LNAs) that target these miRNAs suppress melanoma metastasis [73]. It has been shown that miR-25 levels are higher in melanoma cell lines in comparison to melanocytes, and a miR-25 inhibitor decreases cell migration and proliferation [111]. The transcript of DKK3 (Dickkopf-related protein 3) is a direct target of miR-25, which indicates an important role of miR-25 in the regulation of the WNT/β-catenin pathway. DKK3 inhibition in melanoma cells results in the upregulation of nuclear β-catenin, whereas upregulation of DKK3 partially attenuates the oncogenic effects of miR-25 in melanoma cells. Moreover, ectopic expression of miR-25 in melanoma cells inhibits the activity of TCF4, cyclin D1, and c-Myc [111].

miR-17, which is upregulated in melanoma, increases the motility and migration of melanoma cells. miR-17 is a member of the oncogenic miR-17/92 cluster that targets ETV1 (E twenty-six variant 1) mRNA, which belongs to the ETS (E-twenty-six) family of transcription factors that exert a suppressive role in melanoma [109].

## 6. miRNAs Modulate the Melanoma Metastatic Niche

**Figure 2:**
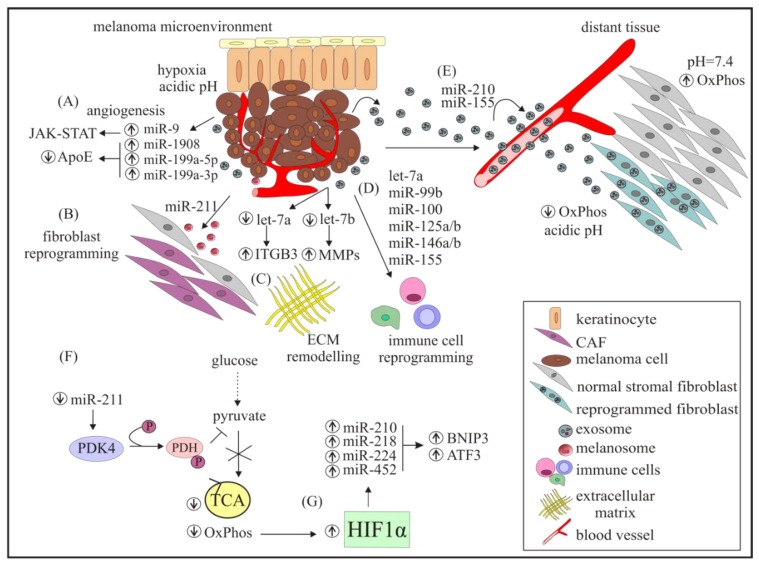
Characteristics of melanoma microenvironment modifications triggered by miRNAs, and the formation of a metastatic niche by melanoma-derived exosomes. The melanoma microenvironment is comprised of various types of cells, and modified by miRNAs that play an important role in: (**A**) angiogenesis; (**B**) transformation of normal fibroblasts toward CAFs (cancer-associated fibroblasts); (**C**) ECM remodeling; and (**D**) immune cell reprogramming. (**F**) Modulation of metabolic pathways. (**G**) HIF-1α stabilized in hypoxic conditions increases the levels of several miRNAs. (**E**) Melanoma-derived exosomes containing miRNAs that contribute to reprogramming cells in distant tissues to prepare the pre-metastatic niche. AFT3: activating transcription factor 3; BNIP3: BCL2/adenovirus E1B interacting protein 3; ECM: extracellular matrix; HIF-1α: hypoxia inducible factor 1 α; OxPhos: oxidative phosphorylation; PDH: pyruvate dehydrogenase; PDK4: pyruvate dehydrogenase kinase 4; and TCA: tricarboxylic acid cycle.

miRNAs can participate in building a pre-metastatic niche that permits both the implantation of tumour cells into distant organs as well as their survival (Figure 1 and Figure 2). This intercellular communication is enabled primarily by melanoma-derived exosomes that contain miRNAs [139].

In addition to primary tumour-derived exosomes, BMDCs and the local stromal environment of the metastatic organ play an important role in the generation of a pre-metastatic niche [140]. Exosomes mediate the cross-talk between cells and favor pre-metastatic niche formation by “educating” BMDCs toward a pro-metastatic and pro-vasculogenic phenotype [141]. In vivo experiments have proven that pre-education of BMDCs with tumour exosomes from a highly metastatic melanoma is sufficient to accelerate tumour growth. Increase in the size and number of metastases, enhanced recruitment of BMDCs and tumour vascular density are characteristic of BM-educated mice in comparison to controls (PBS-treated mice) [141]. MET signaling usually mediates heterotypic cell–cell interactions [142], and has been identified as being involved in the “education” of BMDCs. Furthermore, the transfer of the MET oncoprotein from tumour-derived exosomes to BM progenitor cells triggers the metastatic process in vivo [141]. Furthermore, the involvement of RAB (Ras-related protein) in the secretion of exosomes has been demonstrated, where *RAB27A* knockdown in melanoma cells reduces exosome production and prevents the recruitment of BMDCs [141]. This melanoma-specific “exosome signature” comprises of TYRP2 (tyrosinase-related protein-2), VLA-4 (very late antigen 4), HSP70 (heat shock protein 70), HSP90 [141], and the MET onocoprotein. miR-triggered modulation of the pre-metastatic niche in distal sites is presented in Figure 2.

## 7. Alterations in miRNAs Expression in Response to Microenvironmental Changes

Tumour cells are characterized by their ability to adapt to various conditions that are crucial for invading surrounding tissue and for seeding distant sites to form metastases. Hypoxia is a common feature of the solid tumour microenvironment. Low levels of oxygen promote the invasive potential of melanoma cells [143]. The mechanisms of cancer cell responses to hypoxia involve changes in miRNA levels [144]. It has been shown that 70 miRNAs are differentially expressed in hypoxic and normoxic conditions, and miR-340 may be responsible for the elevated levels of ABCB5 and several other proteins under low oxygen concentrations [145]. Hypoxia-inducible factor 1 alpha (HIF1α) is the main regulator of the cellular response to oxygen deprivation [146]. HIF1α increases the levels of miR-210, miR-218, miR-224, and miR-452 [147], which may contribute to cell cycle progression. Furthermore, the overexpression of these miRNAs increases the expression of *BNIP3* (BCL2/adenovirus E1B interacting protein 3) and *AFT3* (activating transcription factor 3) [147]. Maes et al. have found that BNIP3 contributes to melanoma cell plasticity and aggressiveness [148]. Moreover, the aggressive melanoma cell phenotype driven by BNIP3 is associated with glutamine metabolism in melanoma cells [149]. ATF3 promotes cancer progression by activating an adaptive program to help cells cope with stress [150]. A low level of oxygen increases the number of individual miRNAs and their overall quantity in exosomes [151]. Comparing miRNA profiles in exosomes derived from melanoma cells grown at high (21%) and low (1%) concentrations of oxygen revealed that 15 miRNAs were significantly more abundant under hypoxic conditions, with miR-494, miR-6087, miR-513a, and miR-4497 being the most dramatically enriched [152].

Melanoma-derived exosomes can reprogram adult dermal fibroblasts by increasing aerobic glycolysis and decreasing oxidative phosphorylation, leading to extracellular acidification [34]. Acidic pH stimulates melanoma cells to form pulmonary metastases in athymic nude mice [153], and high levels of VEGF-α, MMP-2, MMP-9, IL-8, cathepsin B, and cathepsin L have been detected under these conditions [153]. Interestingly, as acidic pH induces the release of exosomes, buffering acidic tumour microenvironments decreases the efficiency of this process [154].

Shu et al. have observed that tumour-derived exosomes modulate adult dermal fibroblasts, causing increased glycolysis and decreased oxidative phosphorylation [34]. miR-155 and miR-210 identified in melanoma-derived exosomes favor glycolysis, which simultaneously dampens oxidative phosphorylation and supports the Warburg effect [34]. miR-211 that is involved in the Warburg effect allows melanoma cells to survive under low oxygen pressure. Decreased *miR-211* expression has been observed in melanoma cells in comparison to melanocytes [124]. PDK4 (pyruvate dehydrogenase kinase 4) mRNA has been identified as a miR-211 target. As decreased *miR-211* expression in melanoma elevates PDK4 levels, which in turn inhibit PDH (pyruvate dehydrogenase) via phosphorylation, pyruvate is rerouted away from TCA (tricarboxylic acid cycle), and therefore limits oxidative phosphorylation. Reduced mitochondrial activity decreases oxygen consumption and leads to increased HIF-1α stability. Contrary to melanoma cells, melanocytes maintain a normal oxidative metabolic state [124]. The interplay between miRs and metabolic pathways in melanoma cells is presented in Figure 2.

## 8. miRNAs in Drug Resistance to Targeted Therapy and Immunotherapy

Recently developed therapeutics that target oncoproteins crucial to melanoma development, such as BRAF^V600^, brought hope for patients with metastatic melanoma. However, the development of resistance almost inevitably leads to patient relapse [12,155]. Several mechanisms involved in resistance to BRAF inhibitors (BRAFi) have been identified in melanoma [13,156,157]. miRNA involvement in the development of resistance to BRAFi has been shown [122,158,159,160,161,162,163]. It has been demonstrated in clinical samples that a low level of miR-200c correlates with the development of resistance to BRAFi [122]. The resistance to BRAFi could be reversed by miR-7, which targets EGFR/IGF-1R/CRAF and inhibits the MAPK and PI3K/AKT signaling pathways [161]. It has been shown that miR-514a overexpression in melanoma cell lines inhibits NF1 expression, which correlates with an increased survival of BRAF^V600E^ cells treated with vemurafenib [162]. miR-579, that acts as an oncosuppressor, has also been recognized as an antagonist of drug resistance [159]. Enforced expression of *miR-579* reverts drug resistance to BRAFi in combination with MEKi, and more interestingly, prevents the development of acquired drug resistance. As *miR-579* is located in a MITF-responsive gene encoding ZFR (zink-finger recombinase), its expression is probably dependent on MITF activity. Therefore, a reduced level of MITF in drug resistant cells might be responsible for the downregulation of miR-579 [159]. High expression levels of *miR-100* and *miR-125b* have been detected in vitro in resistant cells and in tumour biopsies obtained from treated patients [163], and their inhibition restores drug efficacy in resistant melanoma cells. Analysis of small RNAseq data has shown higher levels of miR-204 and miR-211 in BRAFi resistant melanoma cells in comparison to their drug-naïve counterparts [160]. This effect has been detected not only in response to BRAFi but also in response to inhibitors of other elements of the MAPK pathway. These two miRNAs have been previously recognized as tumour suppressors that inhibit melanoma cell invasion [99,102,124,164].

Interestingly, miRNAs may also be associated with melanoma resistance to treatment with immune checkpoint inhibitors (ICIs) [165]. A panel of circulating miRNAs (let-7e, miR-99b, miR-100, miR-125a, miR-125b, miR-146a, miR-146b, and miR-155) associated with the activity of MDSC (myeloid-derived suppressor cells) has been identified in melanoma patients [165]. These miRNAs had been recognized as being involved in myeloid cell differentiation and polarization [166,167]. They seem to be responsible for the conversion of monocytes into MDSCs, mediated by melanoma EVs, and their baseline levels correlate with the clinical efficacy of ICIs. Preclinical data has shown that tumour resistance to ICI blockade can be reverted by myeloid cell depletion [168]. miR-146 has been shown to promote M2 polarization [169], and exert a negative feedback on the activation of NF-κB–related genes mediated by TLR4 (Toll-like receptor 4) [170]. miR-125a and miR-125b have been reported to be involved in monocyte differentiation toward immunosuppressive phenotypes [169]. The miRNA-99b/let-7e/miRNA-125a cluster has been shown to stabilize the activity of STAT-3 in tolerogenic APCs (antigen-presenting cells) [171]. Thus, miRNAs may be considered in new therapeutic strategies that seek to overcome BRAFi and ICI resistance.

## 9. miRNAs as Biomarkers and Therapeutics

There is a great need for new diagnostic tools to detect melanoma, especially at the early curable stages of development. miRNAs have great potential as biomarkers because they can discriminate among diverse types of cancers and they are chemically stable and resistant to RNase activity. Fleming et al., have demonstrated that the assessment of four miRNAs (miR-15b, miR-30d, miR-150, and miR-425) has a greater potential of predicting melanoma recurrence than TNM staging [172]. Shiiyama et al. identified six miRNAs (miR-9, miR-145, miR-150, miR-155, miR-203, and miR-205) that were differentially expressed in metastatic melanoma patients compared to healthy donors, and their combination was more sensitive for detecting metastasis than each miRNA assessed individually [173]. The comparison of miRNAs from melanoma tissue samples with matching serum samples revealed that several miRNAs (e.g., miR-221, miR-222, and miR-3201) are only present at high levels in serum, whereas others are exclusively tissue e-derived (e.g., miR-30 and miR-374) [174,175,176]. As melanoma survival rates are higher when the cancer is diagnosed early, non-invasive liquid biopsies would be an optimal source of biomarkers that indicate disease development. This approach may also provide an opportunity for disease monitoring during treatment. In this regard, miRNAs are a long-investigated component of the circulating transcriptome [177,178]. In a recent study, 11 miRNAs (let-7b, miR-16, miR-21, miR-92b, miR-98, miR-134, miR-320a, miR-486, miR-628, miR1180, and miR-1827) were identified as differentially expressed between healthy controls and plasma samples from different melanoma stages [179]. Two miRNAs (miR-320a and miR-134) have been found at lower levels in plasma from melanoma patients compared to samples from healthy donors [179]. miR-21 has been reported at elevated levels in melanoma plasma samples [180]. Eight miRNAs (let-7e, miR-99b, miR-100, miR-125a, miR-125b, miR-146a, miR-146b, and miR-155) have been detected in patients receiving the ICIs ipilimumab and nivolumab as correlated with the frequency of altered myeloid cells and shorter PFS (progression-free survival) and OS (overall survival) [165].

Additional studies are needed for a more critical evaluation of the clinical value of miRNAs, and the use of miRNAs’ expression patterns as reliable biomarkers capable of detecting primary disease and early metastasis after treatment.

From the perspective of therapeutic applications, strategies to deliver tumour-suppressive miRNAs or interfere with tumour-promoting miRNAs are still under development [181,182,183,184].

## 10. Conclusions

Despite recent advances in cancer treatment, metastasis remains the main cause of cancer-related deaths. The findings reviewed here indicate the involvement of miRNAs in melanoma metastasis. Notably, diminished expression of tumour suppressor miRNAs and overexpression of oncomiRs is observed in all stages of melanoma progression, from the non-invasive state to the stage of distant organ colonization. Expression of miRNAs is altered by genomic aberrations, point mutations, chromosomal deletions, and aberrant promoter methylation, however, the expression of numerous miRNAs linked to cancer is also modulated by the microenvironment. Considering the role of miRNAs in melanoma development, miRNAs or their antagonists may be considered for potential therapeutic applications, however, the strategies for this approach are still under development. Additional studies are needed to establish the relationship between miRNAs and the molecular pathways involved in melanoma. Distinct profiles of miRNA expression are detected at each step of melanoma development, and altered expression of miRNAs has been found to correlate with poor prognosis and insufficient response to treatment. Therefore, miRNAs, especially those being part of the circulating transcriptome may be useful as biomarkers for early melanoma detection and response to treatment.

## Figures and Tables

**Table 1:** Invasiveness-associated miRNAs, exhibiting either upregulated (oncomiRs) or downregulated (tumour suppressor miRs) expression in melanoma.

**Upregulated miRNAs**				
**miRNA**	**Mechanism of miRNA Regulation Recognized/Confirmed in Melanoma**	**Target** **mRNA**	**Type of Experiment**	**References**
miR-17	ND	ETV1	in vitro	[106]
miR-19	ND	PITX1	in vitro	[107]
			clinical specimens	
miR-21	AP-1/c-Jun	TIMP3	in vitro	[108]
			in vivo	
		PTEN	in vitro	[109]
			clinical specimens	
		PDCD4 FBXO11	in vitro	[110]
			in vivo	
			clinical specimens	
miR-25	ND	DKK3	in vitro	[111]
			in vivo	
			clinical specimens	
		RBM47	in vitro	[112]
miR-30d/30b	ND	GALNT7	in vitro	[84]
			in vivo	
			clinical specimens	
miR-125b	TCF-4	NEDD9	in vitro	[113]
			clinical specimens	
miR-146a	MYC	NUMB	in vitro	[114]
			in vivo	
			clinical specimens	
miR-182	methylation [97]	MITF-M FOXO3	in vitro	[96]
			in vivo	
			clinical specimens	
		APC	in vitro	[14]
			in vivo	
			clinical specimens	
miR-214	ND	AP-2 γ	in vitro	[75]
			in vivo	
		ITGA3	clinical specimens	
miR-221/222	PLZF	c-KIT	in vitro	[68,69]
		P27	in vivo	
			in vitro	[70]
	MITF		in silico	[115]
miR-224 miR-452	E2F1	TXNIP	in vitro	[116]
			in vivo	
			clinical specimens	
miR-340	CRD-BP	MITF	in vitro	[105]
miR-638	AP-2α	TP53INP2	in vitro	[117]
			in vivo	
			clinical specimens	
miR-1908 miR-199a-5p miR-199a-3p	LNAs	ApoE DNAJA4	in vitro	[73]
			in vivo	
			clinical specimens	
**Downregulated miRNAs**				
**miRNA**	**Mechanism of miRNA Regulation Recognized/Confirmed in Melanoma**	**Target mRNA**	**Type of Experiment**	**References**
let-7a	ND	ITGB3	in vitro	[118]
			clinical specimens	
let-7b	ND	BSG	in vitro	[119]
			in vivo	
miR-34b miR-34c	methylation	MET	in vitro	[120,121]
miR-137	*α-MSH*	PIK3R3	in vitro	[94]
			clinical specimens	
		*MITF*	in vitro	[92]
miR-200c	ND	*BMI-1*	in vitro	[122]
			in vivo	
			clinical specimens	
miR-203	methylation	SLUG IL-8	in vitro	[71]
			in vivo	
			clinical specimens	
		CREB1	in vitro	[123]
miR-211	methylation	IGF2R TGFBR2 NFAT5	in vitro	[99]
		POU3F2/BRN2	in vitro	[101]
		PDK4	in vitro	[124]
			clinical specimens	
miR-218	ND	CIP2A	in vitro	[125]
		BMI1	clinical specimens	
miR-199a-3p	LNAs	MET	in vitro	[120]
miR-675	ND	MTDH	in vitro	[126]
			in vivo	
			clinical specimens	

ND: not determined.

## References

[1] Adler N.R., Haydon A., McLean C.A., Kelly J.W., Mar V.J. (2017). Metastatic pathways in patients with cutaneous melanoma. Pigment Cell Melanoma Res..

[2] Hayward N.K., Wilmott J.S., Waddell N., Johansson P.A., Field M.A., Nones K., Patch A.M., Kakavand H., Alexandrov L.B., Burke H. (2017). Whole-genome landscapes of major melanoma subtypes. Nature.

[3] Alexandrov L.B., Nik-Zainal S., Wedge D.C., Aparicio S.A., Behjati S., Biankin A.V., Bignell G.R., Bolli N., Borg A., Børresen-Dale A.L. (2013). Signatures of mutational processes in human cancer. Nature.

[4] Liu W., Kelly J.W., Trivett M., Murray W.K., Dowling J.P., Wolfe R., Mason G., Magee J., Angel C., Dobrovic A. (2007). Distinct clinical and pathological features are associated with the BRAF(T1799A(V600E)) mutation in primary melanoma. J. Investig. Dermatol..

[5] Long G.V., Stroyakovskiy D., Gogas H., Levchenko E., de Braud F., Larkin J., Garbe C., Jouary T., Hauschild A., Grob J.J. (2014). Combined BRAF and MEK inhibition versus BRAF inhibition alone in melanoma. N. Engl. J. Med..

[6] Rizos H., Menzies A.M., Pupo G.M., Carlino M.S., Fung C., Hyman J., Haydu L.E., Mijatov B., Becker T.M., Boyd S.C. (2014). BRAF inhibitor resistance mechanisms in metastatic melanoma: Spectrum and clinical impact. Clin. Cancer Res..

[7] Gatzka M.V. (2018). Targeted Tumor Therapy Remixed-An Update on the Use of Small-Molecule Drugs in Combination Therapies. Cancers (Basel).

[8] Larkin J., Hodi F.S., Wolchok J.D. (2015). Combined Nivolumab and Ipilimumab or Monotherapy in Untreated Melanoma. N. Engl. J. Med..

[9] Robert C., Schachter J., Long G.V., Arance A., Grob J.J., Mortier L., Daud A., Carlino M.S., McNeil C., Lotem M. (2015). KEYNOTE-006 investigators. Pembrolizumab versus Ipilimumab in Advanced Melanoma. N. Engl. J. Med..

[10] Tucci M., Passarelli A., Mannavola F., Stucci L.S., Ascierto P.A., Capone M., Madonna G., Lopalco P., Silvestris F. (2017). Serum exosomes as predictors of clinical response to ipilimumab in metastatic melanoma. Oncoimmunology.

[11] Luke J.J., Flaherty K.T., Ribas A., Long G.V. (2017). Targeted agents and immunotherapies: Optimizing outcomes in melanoma. Nat. Rev. Clin. Oncol..

[12] Kaufman H.L., Margolin K., Sullivan R. (2018). Management of Metastatic Melanoma in 2018. JAMA Oncol..

[13] Ahmed F., Haass N.K. (2018). Microenvironment-driven dynamic heterogeneity and phenotypic plasticity as a mechanism of melanoma therapy resistance. Front. Oncol..

[14] Croce C.M. (2009). Causes and consequences of microRNA dysregulation in cancer. Nat. Rev. Genet..

[15] Chan S.H., Wang L.H. (2015). Regulation of cancer metastasis by microRNAs. J. Biomed. Sci..

[16] Jing Z., Han W., Sui X., Xie J., Pan H. (2015). Interaction of autophagy with microRNAs and their potential therapeutic implications in human cancers. Cancer Lett..

[17] Su Y., Li X., Ji W., Sun B., Xu C., Li Z., Qian G., Su C. (2014). Small molecule with big role: MicroRNAs in cancer metastatic microenvironments. Cancer Lett..

[18] Chan B., Manley J., Lee J., Singh S.R. (2015). The emerging roles of microRNAs in cancer metabolism. Cancer Lett..

[19] Melnik B.C. (2015). MiR-21: An environmental driver of malignant melanoma?. J. Transl. Med..

[20] Bennett P.E., Bemis L., Norris D.A., Shellman Y.G. (2013). MiR in melanoma development: MiRNAs and acquired hallmarks of cancer in melanoma. Physiol. Genom..

[21] Varamo C., Occelli M., Vivenza D., Merlano M., Lo Nigro C. (2017). MicroRNAs role as potential biomarkers and key regulators in melanoma. Genes Chromosomes Cancer.

[22] Mirzaei H., Gholamin S., Shahidsales S., Sahebkar A., Jaafari M.R., Mirzaei H.R., Hassanian S.M., Avan A. (2016). MicroRNAs as potential diagnostic and prognostic biomarkers in melanoma. Eur. J. Cancer.

[23] Wozniak M., Mielczarek A., Czyz M. (2016). MiRNAs in melanoma: Tumor suppressors and oncogenes with prognostic potential. Curr. Med. Chem..

[24] Mione M., Bosserhoff A. (2015). MicroRNAs in melanocyte and melanoma biology. Pigment Cell Melanoma Res..

[25] Mannavola F., Tucci M., Felici C., Stucci S., Silvestris F. (2016). MiRNAs in melanoma: A defined role in tumor progression and metastasis. Expert Rev. Clin. Immunol..

[26] Mueller D.W., Rehli M., Bosserhoff A.K. (2009). MiRNA expression profiling in melanocytes and melanoma cell lines reveals miRNAs associated with formation and progression of malignant melanoma. J. Investig. Dermatol..

[27] Li J.Y., Zheng L.L., Wang T.T., Hu M. (2016). Functional annotation of metastasis-associated microRNAs of melanoma: A meta-analysis of expression profiles. Chin. Med. J. (Engl.).

[28] Caramuta S., Egyházi S., Rodolfo M., Witten D., Hansson J., Larsson C., Lui W.O. (2010). MicroRNA expression profiles associated with mutational status and survival in malignant melanoma. J. Investig. Dermatol..

[29] Chen P.S., Su J.L., Hung M.C. (2012). Dysregulation of microRNAs in cancer. J. Biomed. Sci..

[30] Couts K.L., Anderson E.M., Gross M.M., Sullivan K., Ahn N.G. (2013). Oncogenic B-Raf signaling in melanoma cells controls a network of microRNAs with combinatorial functions. Oncogene.

[31] Latchana N., Abrams Z.B., Howard J.H., Regan K., Jacob N., Fadda P., Terando A., Markowitz J., Agnese D., Payne P. (2017). Plasma microRNA levels following resection of metastatic melanoma. Bioinform. Biol. Insights.

[32] Mo M.H., Chen L., Fu Y., Wang W., Fu S.W. (2012). Cell-free Circulating miRNA Biomarkers in Cancer. J. Cancer.

[33] Dror S., Sander L., Schwartz H., Sheinboim D., Barzilai A., Dishon Y., Apcher S., Golan T., Greenberger S., Barshack I. (2016). Melanoma miRNA trafficking controls tumour primary niche formation. Nat. Cell Biol..

[34] La Shu S., Yang Y., Allen C.L., Maguire O., Minderman H., Sen A., Ciesielski M.J., Collins K.A., Bush P.J., Singh P. (2018). Metabolic reprogramming of stromal fibroblasts by melanoma exosome microRNA favours a pre-metastatic microenvironment. Sci. Rep..

[35] Lee R.C., Feinbaum R.L., Ambros V. (1993). The, C. elegans heterochronic gene lin-4 encodes small RNAs with antisense complementarity to lin-14. Cell.

[36] Bhaskaran M., Mohan M. (2014). MicroRNAs: History, biogenesis, and their evolving role in animal development and disease. Vet. Pathol..

[37] Davalos V., Esteller M. (2010). MicroRNAs and cancer epigenetics: A macrorevolution. Curr. Opin. Oncol..

[38] Fabbri M., Calore F., Paone A., Galli R., Calin G.A. (2013). Epigenetic regulation of miRNAs in cancer. Adv. Exp. Med. Biol..

[39] Shen J., Xia W., Khotskaya Y.B., Huo L., Nakanishi K., Lim S.O., Du Y., Wang Y., Chang W.C., Chen C.H. (2013). EGFR modulates microRNA maturation in response to hypoxia through phosphorylation of AGO2. Nature.

[40] Bartel D.P. (2009). MicroRNAs: Target recognition and regulatory functions. Cell.

[41] Kim D., Sung Y.M., Park J., Kim S., Kim J., Park J., Ha H., Bae J.Y., Kim S., Baek D. (2016). General rules for functional microRNA targeting. Nat. Genet..

[42] Thomson D.W., Dinger M.E. (2016). Endogenous microRNA sponges: Evidence and controversy. Nat. Rev. Genet..

[43] Hansen T.B., Jensen T., Clausen B.H., Bramsen J.B., Finsen B., Damgaard C.K., Kjems J. (2013). Natural RNA circles function as efficient microRNA sponges. Nature.

[44] Poliseno L., Salmena L., Zhang J., Carver B., Haveman W.J., Pandolfi P.P. (2010). A coding-independent function of gene and pseudogene mRNAs regulates tumour biology. Nature.

[45] Gilot D., Migault M., Bachelot L., Journé F., Rogiers A., Donnou-Fournet E., Mogha A., Mouchet N., Pinel-Marie M.L., Mari B. (2017). A non-coding function of TYRP1 mRNA promotes melanoma growth. Nat. Cell Biol..

[46] Hartman M.L., Czyz M. (2019). TYRP1 mRNA level is stable and MITF-M-independent in drug-naïve and vemurafenib-resistant melanoma cells. Melanoma Res..

[47] Maffioletti E., Tardito D., Gennarelli M., Bocchio-Chiavetto L. (2014). Micro spies from the brain to the periphery: New clues from studies on microRNAs in neuropsychiatric disorders. Front. Cell. Neurosci..

[48] Sheervalilou R., Shirvaliloo S., Aval S.F., Khamaneh A.M., Sharifi A., Ansarin K., Zarghami N. (2017). A new insight on reciprocal relationship between microRNA expression and epigenetic modifications in human lung cancer. Tumor Biol..

[49] Jahagirdar D., Purohit S., Jain A., Sharma N.K. (2016). Export of microRNAs: A Bridge between Breast Carcinoma and Their Neighboring Cells. Front. Oncol..

[50] Minciacchi V.R., Freeman M.R., Di Vizio D. (2015). Extracellular vesicles in cancer: Exosomes, microvesicles and the emerging role of large oncosomes. Semin. Cell Dev. Biol..

[51] Becker A., Thakur B.K., Weiss J.M., Kim H.S., Peinado H., Lyden D. (2016). Extracellular Vesicles in Cancer: Cell-to-Cell Mediators of Metastasis. Cancer Cell.

[52] Di Vizio D., Morello M., Dudley A.C., Schow P.W., Adam R.M., Morley S., Mulholland D., Rotinen M., Hager M.H., Insabato L. (2012). Large oncosomes in human prostate cancer tissues and in the circulation of mice with metastatic disease. Am. J. Pathol..

[53] Plotkin L.I., Pacheco-Costa R., Davis H.M. (2017). microRNAs and connexins in bone: Interaction and mechanisms of delivery. Curr. Mol. Biol. Rep..

[54] Kosaka N., Iguchi H., Ochiya T. (2010). Circulating microRNA in body fluid: A new potential biomarker for cancer diagnosis and prognosis. Cancer Sci..

[55] Huang X., Yuan T., Tschannen M., Sun Z., Jacob H., Du M., Liang M., Dittmar R.L., Liu Y., Liang M. (2013). Characterization of human plasma-derived exosomal RNAs by deep sequencing. BMC Genom..

[56] Al-Nedawi K., Meehan B., Micallef J., Lhotak V., May L., Guha A., Rak J. (2008). Intercellular transfer of the oncogenic receptor EGFRvIII by microvesicles derived from tumour cells. Nat. Cell Biol..

[57] Pan B.T., Johnstone R.M. (1983). Fate of the transferrin receptor during maturation of sheep reticulocytes in vitro: Selective externalization of the receptor. Cell.

[58] Raposo G., Stoorvogel W. (2013). Extracellular vesicles: Exosomes, microvesicles, and friends. J. Cell Biol..

[59] Colombo M., Moita C., van Niel G., Kowal J., Vigneron J., Benaroch P., Manel N., Moita L.F., Théry C., Raposo G. (2013). Analysis of ESCRT functions in exosome biogenesis, composition and secretion highlights the heterogeneity of extracellular vesicles. J. Cell Sci..

[60] Shimaoka M., Kawamoto E., Gaowa A., Okamoto T., Park E.J. (2019). Connexins and Integrins in Exosomes. Cancers.

[61] Jella K.K., Nasti T.H., Li Z., Malla S.R., Buchwald Z.S., Khan M.K. (2018). Exosomes, Their Biogenesis and Role in Inter-Cellular Communication, Tumor Microenvironment and Cancer Immunotherapy. Vaccines (Basel).

[62] Gajos-Michniewicz A., Duechler M., Czyz M. (2014). MiRNA in melanoma-derived exosomes. Cancer Lett..

[63] Tucci M., Mannavola F., Passarelli A., Stucci L.S., Cives M., Silvestris F. (2018). Exosomes in melanoma: A role in tumor progression, metastasis and impaired immune system activity. Oncotarget.

[64] Grasedieck S., Sorrentino A., Langer C., Buske C., Döhner H., Mertens D., Kuchenbauer F. (2013). Circulating microRNAs in hematological diseases: Principles, challenges, and perspectives. Blood.

[65] Boissy R.E. (2003). Melanosome transfer to and translocation in the keratinocyte. Exp. Dermatol..

[66] Golan T., Messer A.R., Amitai-Lange A., Melamed Z., Ohana R., Bell R.E., Kapitansky O., Lerman G., Greenberger S., Khaled M. (2015). Interactions of melanoma cells with distal keratinocytes trigger metastasis via Notch signaling inhibition of MITF. Mol. Cell.

[67] Felli N., Errico M.C., Pedini F., Petrini M., Puglisi R., Bellenghi M., Boe A., Felicetti F., Mattia G., De Feo A. (2016). AP2α controls the dynamic balance between miR-126&126* and miR-221&222 during melanoma progression. Oncogene.

[68] Felicetti F., Errico M.C., Bottero L., Segnalini P., Stoppacciaro A., Biffoni M., Felli N., Mattia G., Petrini M., Colombo M.P. (2008). The promyelocytic leukemia zinc finger-microRNA-221/-222 pathway controls melanoma progression through multiple oncogenic mechanisms. Cancer Res..

[69] Felicetti F., De Feo A., Coscia C., Puglisi R., Pedini F., Pasquini L., Bellenghi M., Errico M.C., Pagani E., Carè A. (2016). Exosome-mediated transfer of miR-222 is sufficient to increase tumor malignancy in melanoma. J. Transl. Med..

[70] Igoucheva O., Alexeev V. (2009). MicroRNA-dependent regulation of cKit in cutaneous melanoma. Biochem. Biophys. Res. Commun..

[71] Lohcharoenkal W., Das Mahapatra K., Pasquali L., Crudden C., Kular L., Akkaya Ulum Y.Z., Zhang L., Xu Landén N., Girnita L., Jagodic M. (2018). Genome-Wide Screen for MicroRNAs Reveals a Role for miR-203 in Melanoma Metastasis. J. Investig. Dermatol..

[72] Singh R.K., Varney M.L. (2000). IL-8 expression in malignant melanoma: Implications in growth and metastasis. Histol. Histopathol..

[73] Pencheva N., Tran H., Buss C., Huh D., Drobnjak M., Busam K., Tavazoie S.F. (2012). Convergent multi-miRNA targeting of ApoE drives LRP1/LRP8 dependent melanoma metastasis and angiogenesis. Cell.

[74] Zhuang G., Wu X., Jiang Z., Kasman I., Yao J., Guan Y., Oeh J., Modrusan Z., Bais C., Sampath D. (2012). Tumour-secreted miR-9 promotes endothelial cell migration and angiogenesis by activating the JAK-STAT pathway. EMBO J..

[75] Penna E., Orso F., Cimino D., Tenaglia E., Lembo A., Quaglino E., Poliseno L., Haimovic A., Osella-Abate S., De Pittà C. (2011). MicroRNA-214 contributes to melanoma tumour progression through suppression of TFAP2C. EMBO J..

[76] Crawford Y., Kasman I., Yu L., Zhong C., Wu X., Modrusan Z., Kaminker J., Ferrara N. (2009). PDGF-C mediates the angiogenic and tumorigenic properties of fibroblasts associated with tumors refractory to anti-VEGF treatment. Cancer Cell.

[77] Anderberg C., Li H., Fredriksson L., Andrae J., Betsholtz C., Li X., Eriksson U., Pietras K. (2009). Paracrine signaling by platelet-derived growth factor-CC promotes tumor growth by recruitment of cancer-associated fibroblasts. Cancer Res..

[78] Di Tomaso E., London N., Fuja D., Logie J., Tyrrell J.A., Kamoun W., Munn L.L., Jain R.K. (2009). PDGF-C induces maturation of blood vessels in a model of glioblastoma and attenuates the response to anti-VEGF treatment. PLoS ONE.

[79] Passarelli A., Mannavola F., Stucci L.S., Tucci M., Silvestris F. (2017). Immune system and melanoma biology: A balance between immunosurveillance and immune escape. Oncotarget.

[80] Fanini F., Fabbri M. (2017). Cancer-derived exosomic microRNAs shape the immune system within the tumor microenvironment: State of the art. Semin. Cell Dev. Biol..

[81] Graner M.W., Schnell S., Olin M.R. (2018). Tumor-derived exosomes, microRNAs, and cancer immune suppression. Semin. Immunopathol..

[82] Alfonsi R., Grassi L., Signore M., Bonci D. (2018). The Double Face of Exosome-Carried MicroRNAs in Cancer Immunomodulation. Int. J. Mol. Sci..

[83] Vallacchi V., Camisaschi C., Dugo M., Vergani E., Deho P., Gualeni A., Huber V., Gloghini A., Maurichi A., Santinami M. (2016). MicroRNA Expression in Sentinel Nodes from Progressing Melanoma Patients Identifies Networks Associated with Dysfunctional Immune Response. Genes (Basel).

[84] Gaziel-Sovran A., Segura M.F., Di Micco R., Collins M.K., Hanniford D., Vega-Saenz de Miera E., Rakus J.F., Dankert J.F., Shang S., Kerbel R.S. (2011). MiR30b/30d regulation of GalNAc transferases enhances invasion and immunosuppression during metastasis. Cancer Cell.

[85] Ding H., Yang X., Wei Y. (2018). Fusion Proteins of NKG2D/NKG2DL in Cancer Immunotherapy. Int. J. Mol. Sci..

[86] Heinemann A., Zhao F., Pechlivanis S., Eberle J., Steinle A., Diederichs S., Schadendorf D., Paschen A. (2012). Tumor suppressive microRNAs miR-34a/c control cancer cell expression of ULBP2, a stress-induced ligand of the natural killer cell receptor NKG2D. Cancer Res..

[87] Paschen A., Sucker A., Hill B., Moll I., Zapatka M., Nguyen X.D., Sim G.C., Gutmann I., Hassel J., Becker J.C. (2009). Differential clinical significance of individual NKG2D ligands in melanoma: Soluble ULBP2 as an indicator of poor prognosis superior to S100B. Clin. Cancer Res..

[88] Garraway L.A., Widlund H.R., Rubin M.A., Getz G., Berger A.J., Ramaswamy S., Beroukhim R., Milner D.A., Granter S.R., Du J. (2005). Integrative genomic analyses identify MITF as a lineage survival oncogene amplified in malignant melanoma. Nature.

[89] Carreira S., Goodall J., Denat L., Rodriguez M., Nuciforo P., Hoek K.S., Testori A., Larue L., Goding C.R. (2006). Mitf regulation of Dia1 controls melanoma proliferation and invasiveness. Genes Dev..

[90] Bell R.E., Levy C. (2011). The three M’s: Melanoma, microphthalmia-associated transcription factor and microRNA. Pigment Cell Melanoma Res..

[91] Hartman M.L., Czyz M. (2015). MITF in melanoma: Mechanisms behind its expression and activity. Cell Mol. Life Sci..

[92] Bemis L.T., Chen R., Amato C.M., Classen E.H., Robinson S.E., Coffey D.G., Erickson P.F., Shellman Y.G., Robinson W.A.F. (2008). MicroRNA-137 targets microphthalmia-associated transcription factor in melanoma cell lines. Cancer Res..

[93] Luo C., Tetteh P.W., Merz P.R., Dickes E., Abukiwan A., Hotz-Wagenblatt A., Holland-Cunz S., Sinnberg T., Schittek B., Schadendorf D. (2013). MiR-137 inhibits the invasion of melanoma cells through downregulation of multiple oncogenic target genes. J. Investig. Dermatol..

[94] Qi J., Wang W.W., Chen W., Lu W.Y., Shang A.Q. (2018). Mechanism of miR-137 regulating migration and invasion of melanoma cells by targeting PIK3R3gene. J. Cell Biochem..

[95] Haflidadóttir B.S., Bergsteinsdóttir K., Praetorius C., Steingrímsson E. (2010). MiR-148 regulates Mitf in melanoma cells. PLoS ONE.

[96] Segura M.F., Hanniford D., Menendez S., Reavie L., Zou X., Alvarez-Diaz S., Zakrzewski J., Blochin E., Rose A., Bogunovic D. (2009). Aberrant miR-182 expression promotes melanoma metastasis by repressing FOXO3 and microphthalmia-associated transcription factor. Proc. Natl. Acad. Sci. USA.

[97] Liu S., Howell P.M., Riker A.I. (2013). Up-regulation of miR-182 expression after epigenetic modulation of human melanoma cells. Ann. Surg. Oncol..

[98] Jukic D.M., Rao U.N., Kelly L., Skaf J.S., Drogowski L.M., Kirkwood J.M., Panelli M.C. (2010). Microrna profiling analysis of differences between the melanoma of young adults and older adults. J. Transl. Med..

[99] Levy C., Khaled M., Iliopoulos D., Janas M.M., Schubert S., Pinner S., Chen P.H., Li S., Fletcher A.L., Yokoyama S. (2010). Intronic miR-211 assumes the tumor suppressive function of its host gene in melanoma. Mol. Cell.

[100] Mazar J., DeYoung K., Khaitan D., Meister E., Almodovar A., Goydos J., Ray A., Perera R.J. (2010). The regulation of miRNA-211 expression and its role in melanoma cell invasiveness. PLoS ONE.

[101] Boyle G.M., Woods S.L., Bonazzi V.F., Stark M.S., Hacker E., Aoude L.G., Dutton-Regester K., Cook A.L., Sturm R.A., Hayward N.K. (2011). Melanoma cell invasiveness is regulated by miR-211 suppression of the BRN2 transcription factor. Pigment Cell Melanoma Res..

[102] Bell R.E., Khaled M., Netanely D., Schubert S., Golan T., Buxbaum A., Janas M.M., Postolsky B., Goldberg M.S., Shamir R. (2014). Transcription factor/microRNA axis blocks melanoma invasion program by miR-211 targeting NUAK1. J. Investig. Dermatol..

[103] Eccles M.R., He S., Ahn A., Slobbe L., Jeffs A.R., Yoon H.S., Baguley B.C. (2013). MITF and PAX3 Play Distinct Roles in Melanoma Cell Migration; Outline of a “Genetic Switch” Theory Involving MITF and PAX3 in Proliferative and Invasive Phenotypes of Melanoma. Front. Oncol..

[104] Arozarena I., Sanchez-Laorden B., Packer L., Hidalgo-Carcedo C., Hayward R., Viros A., Sahai E., Marais R. (2011). Oncogenic BRAF induces melanoma cell invasion by downregulating the cGMP-specific phosphodiesterase PDE5A. Cancer Cell.

[105] Goswami S., Tarapore R.S., Poenitzsch Strong A.M., TeSlaa J.J., Grinblat Y., Setaluri V., Spiegelman V.S. (2015). MicroRNA-340-mediated degradation of microphthalmia-associated transcription factor (MITF) mRNA is inhibited by coding region determinant-binding protein (CRD-BP). J. Biol. Chem..

[106] Cohen R., Greenberg E., Nemlich Y., Schachter J., Markel G. (2015). MiR17 regulates melanoma cell motility by inhibiting the translation of ETV1. Oncotarget.

[107] Ohira T., Naohiro S., Nakayama Y., Osaki M., Okada F., Oshimura M., Kugoh H. (2015). miR-19b regulates hTERT mRNA expression through targeting PITX1 mRNA in melanoma cells. Sci. Rep..

[108] Martin del Campo S.E., Latchana N., Levine K.M., Grignol V.P., Fairchild E.T., Jaime-Ramirez A.C., Dao T.V., Karpa V.I., Carson M., Ganju A. (2015). MiR-21 enhances melanoma invasiveness via inhibition of tissue inhibitor of metalloproteinases 3 expression: In vivo effects of MiR-21 inhibitor. PLoS ONE.

[109] Saldanha G., Potter L., Lee Y.S., Watson S., Shendge P., Pringle J.H. (2016). MicroRNA-21 expression and its pathogenetic significance in cutaneous melanoma. Melanoma Res..

[110] Yang C.H., Pfeffer S.R., Sims M., Yue J., Wang Y., Linga V.G., Paulus E., Davidoff A.M., Pfeffer L.M. (2015). The oncogenic microRNA-21 inhibits the tumor suppressive activity of FBXO11 to promote tumorigenesis. J. Biol. Chem..

[111] Huo J., Zhang Y., Li R., Wang Y., Wu J., Zhang D. (2016). Upregulated microRNA-25 mediates the migration of melanoma cells by targeting DKK3 through the WNT/β-catenin pathway. Int. J. Mol. Sci..

[112] Jiang Q.Q., Liu W.B. (2018). MiR-25 promotes melanoma progression by regulating RNA binding motif protein 47. Med. Sci. (Paris).

[113] Rambow F., Bechadergue A., Luciani F., Gros G., Domingues M., Bonaventure J., Meurice G., Marine J.C., Larue L. (2016). Regulation of Melanoma Progression through the TCF4/miR-125b/NEDD9 Cascade. J. Investig. Dermatol..

[114] Forloni M., Dogra S.K., Dong Y., Conte D., Ou J., Zhu L.J., Deng A., Mahalingam M., Green M.R., Wajapeyee N. (2014). MiR-146a promotes the initiation and progression of melanoma by activating Notch signaling. eLife.

[115] Ozsolak F., Poling L.L., Wang Z., Liu H., Liu X.S., Roeder R.G., Zhang X., Song J.S., Fisher D.E. (2008). Chromatin structure analyses identify miRNA promoters. Genes Dev..

[116] Knoll S., Fürst K., Kowtharapu B., Schmitz U., Marquardt S., Wolkenhauer O., Martin H., Pützer B.M. (2014). E2F1 induces miR 224/452 expression to drive EMT through TXNIP downregulation. EMBO Rep..

[117] Bhattacharya A., Schmitz U., Raatz Y., Schönherr M., Kottek T., Schauer M., Franz S., Saalbach A., Anderegg U., Wolkenhauer O. (2015). MiR-638 promotes melanoma metastasis and protects melanoma cells from apoptosis and autophagy. Oncotarget.

[118] Müller D.W., Bosserhoff A.K. (2008). Integrin beta 3 expression is regulated by let-7a miRNA in malignant melanoma. Oncogene.

[119] Fu T.Y., Chang C.C., Lin C.T., Lai C.H., Peng S.Y., Ko Y.J., Tang P.C. (2011). Let-7b-mediated suppression of basigin expression and metastasis in mouse melanoma cells. Exp. Cell Res..

[120] Migliore C., Petrelli A., Ghiso E., Corso S., Capparuccia L., Eramo A., Comoglio P.M., Giordano S. (2008). MicroRNAs impair MET-mediated invasive growth. Cancer Res..

[121] Mazar J., Khaitan D., DeBlasio D., Zhong C., Govindarajan S.S., Kopanathi S., Zhang S., Ray A., Perera R.J. (2011). Epigenetic regulation of microRNA genes and the role of miR-34b in cell invasion and motility in human melanoma. PLoS ONE.

[122] Liu S., Tetzlaff M.T., Wang T., Yang R., Xie L., Zhang G., Krepler C., Xiao M., Beqiri M., Xu W. (2015). MiR-200c/Bmi1 axis and epithelial-mesenchymal transition contribute to acquired resistance to BRAF inhibitor treatment. Pigment Cell Melanoma Res..

[123] Noguchi S., Kumazaki M., Mori T., Baba K., Okuda M., Mizuno T., Akao Y. (2016). Analysis of microRNA-203 function in CREB/MITF/RAB27a pathway: Comparison between canine and human melanoma cells. Vet. Comp. Oncol..

[124] Mazar J., Qi F., Lee B., Marchica J., Govindarajan S., Shelley J., Li J.L., Ray A., Perera R.J. (2016). MicroRNA 211 Functions as a Metabolic Switch in Human Melanoma Cells. Mol. Cell Biol..

[125] Wei Y., Du Y., Chen X., Li P., Wang Y., Zang W., Zhao L., Li Z., Zhao G. (2014). Expression patterns of microRNA-218 and its potential functions by targeting CIP2A and BMI1 genes in melanoma. Tumor Biol..

[126] Liu K., Jin J., Rong K., Zhuo L., Li P. (2018). MicroRNA-675 inhibits cell proliferation and invasion in melanoma by directly targeting metadherin. Mol. Med. Rep..

[127] Segura M.F., Greenwald H.S., Hanniford D., Osman I., Hernando E. (2012). MicroRNA and cutaneous melanoma: From discovery to prognosis and therapy. Carcinogenesis.

[128] Gokhale A., Kunder R., Goel A., Sarin R., Moiyadi A., Shenoy A., Mamidipally C., Noronha S., Kannan S., Shirsat N.V. (2010). Distinctive microRNA signature of medulloblastomas associated with the WNT signaling pathway. J. Cancer Res. Ther..

[129] Nishinaka Y., Nishiyama A., Masutani H., Oka S., Ahsan K.M., Nakayama Y., Ishii Y., Nakamura H., Maeda M., Yodoi J. (2004). Loss of thioredoxin-bind- ing protein-2/vitamin D3 up-regulated protein 1 in human T-cell leukemia virus type I-dependent T-cell transformation: Implications for adult T-cell leukemia leukemogenesis. Cancer Res..

[130] Adam L., Zhong M., Choi W., Qi W., Nicoloso M., Arora A., Calin G., Wang H., Siefker-Radtke A., McConkey D. (2009). MiR-200 expression regulates epithelial-to-mesenchymal transition in bladder cancer cells and reverses resistance to epidermal growth factor receptor therapy. Clin. Cancer Res..

[131] Elson-Schwab I., Lorentzen A., Marshall C.J. (2010). MicroRNA-200 family members differentially regulate morphological plasticity and mode of melanoma cell invasion. PLoS ONE.

[132] Wang Z., Li Y., Ahmad A., Azmi A.S., Kong D., Banerjee S., Sarkar F.H. (2010). Targeting miRNAs involved in cancer stem cell and EMT regulation: An emerging concept in overcoming drug resistance. Drug Resist. Updat..

[133] Wellner U., Schubert J., Burk U.C., Schmalhofer O., Zhu F., Sonntag A., Waldvogel B., Vannier C., Darling D., zur Hausen A. (2009). The EMT-activator ZEB1 promotes tumorigenicity by repressing stemness-inhibiting microRNAs. Nat. Cell Biol..

[134] Da Cruz A.T., Jasiulionis M.G. (2012). MiRNAs and Melanoma: How Are They Connected?. Dermatol. Res. Pract..

[135] Romano G., Kwong L.N. (2017). MiRNAs, Melanoma and Microenvironment: An Intricate Network. Int. J. Mol. Sci..

[136] Domingues M.J., Rambow F., Job B., Papon L., Liu W., Larue L., Bonaventure J. (2014). Beta-catenin inhibitor ICAT modulates the invasive motility of melanoma cells. Cancer Res..

[137] Ge X., Lv X., Feng L., Liu X., Gao J., Chen N., Wang X. (2012). Metadherin contributes to the pathogenesis of diffuse large B-cell lymphoma. PLoS ONE.

[138] Mazar J., DeBlasio D., Govindarajan S.S., Zhang S., Perera R.J. (2011). Epigenetic regulation of microRNA-375 and its role in melanoma development in humans. FEBS Lett..

[139] Nogués L., Benito-Martin A., Hergueta-Redondo M., Peinado H. (2018). The influence of tumour-derived extracellular vesicles on local and distal metastatic dissemination. Mol. Asp. Med..

[140] Liu Y., Cao X. (2016). Characteristics and Significance of the Pre-metastatic Niche. Cancer Cell.

[141] Peinado H., Alečković M., Lavotshkin S., Matei I., Costa-Silva B., Moreno-Bueno G., Hergueta-Redondo M., Williams C., García-Santos G., Ghajar C. (2012). Melanoma exosomes educate bone marrow progenitor cells toward a pro-metastatic phenotype through MET. Nat. Med..

[142] Czyz M. (2018). HGF/c-MET signaling in melanocytes and melanoma. Int. J. Mol. Sci..

[143] Hanna S.C., Krishnan B., Bailey S.T., Moschos S.J., Kuan P.F., Shimamura T., Osborne L.D., Siegel M.B., Duncan L.M., O′Brien E.T. (2013). HIF1α and HIF2α independently activate SRC to promote melanoma metastases. J. Clin. Investig..

[144] Nouraee N., Mowla S.J., Calin G.A. (2015). Tracking miRNAs’ footprints in tumor-microenvironment interactions: Insights and implications for targeted cancer therapy. Genes Chromosomes Cancer.

[145] Wozniak M., Sztiller-Sikorska M., Czyz M. (2015). Diminution of miR-340-5p levels is responsible for increased expression of ABCB5 in melanoma cells under oxygen-deprived conditions. Exp. Mol. Pathol..

[146] Majmundar A.J., Wong W.J., Simon M.C. (2010). Hypoxia-inducible factors and the response to hypoxic stress. Mol. Cell.

[147] Hwang H.W., Baxter L.L., Loftus S.K., Cronin J.C., Trivedi N.S., Borate B., Pavan W.J. (2014). Distinct microRNA expression signatures are associated with melanoma subtypes and are regulated by HIF1A. Pigment Cell Melanoma Res..

[148] Maes H., Van Eygen S., Krysko D.V., Vandenabeele P., Nys K., Rillaerts K., Garg A.D., Verfaillie T., Agostinis P. (2014). BNIP3 supports melanoma cell migration and vasculogenic mimicry by orchestrating the actin cytoskeleton. Cell Death Dis..

[149] Vara-Perez M., Maes H., Van Dingenen S., Agostinis P. (2019). BNIP3 contributes to the glutamine-driven aggressive behavior of melanoma cells. Biol. Chem..

[150] Thompson M.R., Xu D., Williams B.R.G. (2009). ATF3 transcription factor and its emerging roles in immunity and cancer. J. Mol. Med..

[151] King H.W., Michael M.Z., Gleadle J.M. (2012). Hypoxic enhancement of exosome release by breast cancer cells. BMC Cancer.

[152] Wozniak M., Peczek L., Czernek L., Düchler M. (2017). Analysis of the miRNA profiles of melanoma exosomes derived under normoxic and hypoxic culture conditions. Anticancer Res..

[153] Rofstad E.K., Mathiesen B., Kindem K., Galappathi K. (2006). Acidic extracellular pH promotes experimental metastasis of human melanoma cells in athymic nude mice. Cancer Res..

[154] Logozzi M., Mizzoni D., Angelini D.F., Di Raimo R., Falchi M., Battistini L., Fais S. (2018). Microenvironmental pH and Exosome Levels Interplay in Human Cancer Cell Lines of Different Histotypes. Cancers (Basel).

[155] Wagle N., Van Allen E.M., Treacy D.J., Frederick D.T., Cooper Z.A., Taylor-Weiner A., Rosenberg M., Goetz E.M., Sullivan R.J., Farlow D.N. (2014). MAP kinase pathway alterations in BRAF-mutant melanoma patients with acquired resistance to combined RAF/MEK inhibition. Cancer Discov..

[156] Amaral T., Sinnberg T., Meier F., Krepler C., Levesque M., Niessner H., Garbe C. (2017). The mitogen-activated protein kinase pathway in melanoma part I—Activation and primary resistance mechanisms to BRAF inhibition. Eur. J. Cancer.

[157] Larribère L., Kuphal S., Sachpekidis C., Sachindra, Hüser L., Bosserhoff A., Utikal J. (2018). Targeted Therapy-Resistant Melanoma Cells Acquire Transcriptomic Similarities with Human Melanoblasts. Cancers (Basel).

[158] Migliore C., Giordano S. (2013). Resistance to targeted therapies: A role for microRNAs?. Trends Mol. Med..

[159] Fattore L., Mancini R., Acunzo M., Romano G., Laganà A., Pisanu M.E., Malpicci D., Madonna G., Mallardo D., Capone M. (2016). MiR-579-3p controls melanoma progression and resistance to target therapy. Proc. Natl. Acad. Sci. USA.

[160] Díaz-Martínez M., Benito-Jardón L., Alonso L., Koetz-Ploch L., Hernando E., Teixidó J. (2018). MiR-204-5p and miR-211-5p Contribute to BRAF Inhibitor Resistance in Melanoma. Cancer Res..

[161] Sun X., Li J., Sun Y., Zhang Y., Dong L., Shen C., Yang L., Yang M., Li Y., Shen G. (2016). MiR-7 reverses the resistance to BRAFi in melanoma by targeting EGFR/IGF-1R/CRAF and inhibiting the MAPK and PI3K/AKT signaling pathways. Oncotarget.

[162] Stark M.S., Bonazzi V.F., Boyle G.M., Palmer J.M., Symmons J., Lanagan C.M., Schmidt C.W., Herington A.C., Ballotti R., Pollock P.M. (2015). MiR-514a regulates the tumour suppressor NF1 and modulates BRAFi sensitivity in melanoma. Oncotarget.

[163] Vergani E., Di Guardo L., Dugo M., Rigoletto S., Tragni G., Ruggeri R., Perrone F., Tamborini E., Gloghini A., Arienti F. (2016). Overcoming melanoma resistance to vemurafenib by targeting CCL2-induced miR-34a, miR-100 and miR-125b. Oncotarget.

[164] Luan W., Qian Y., Ni X., Bu X., Xia Y., Wang J., Ruan H., Ma S., Xu B. (2017). MiR-204-5p acts as a tumor suppressor by targeting matrix metalloproteinases-9 and B-cell lymphoma-2 in malignant melanoma. OncoTargets Ther..

[165] Huber V., Vallacchi V., Fleming V., Hu X., Cova A., Dugo M., Shahaj E., Sulsenti R., Vergani E., Filipazzi P. (2018). Tumor-derived microRNAs induce myeloid suppressor cells and predict immunotherapy resistance in melanoma. J. Clin. Investig..

[166] Cobos Jiménez V., Bradley E.J., Willemsen A.M., van Kampen A.H., Baas F., Kootstra N.A. (2014). Next-generation sequencing of microRNAs uncovers expression signatures in polarized macrophages. Physiol. Genom..

[167] Eigsti R.L., Sudan B., Wilson M.E., Graff J.W. (2014). Regulation of activation-associated microRNA accumulation rates during monocyte-to-macrophage differentiation. J. Biol. Chem..

[168] Kaneda M.M., Messer K.S., Ralainirina N., Li H., Leem C.J., Gorjestani S., Woo G., Nguyen A.V., Figueiredo C.C., Foubert P. (2016). PI3Kγ is a molecular switch that controls immune suppression. Nature.

[169] Lee H.M., Kim T.S., Jo E.K. (2016). MiR-146 and miR-125 in the regulation of innate immunity and inflammation. BMB Rep..

[170] Taganov K.D., Boldin M.P., Chang K.J., Baltimore D. (2006). NF-kappaB-dependent induction of microRNA miR-146, an inhibitor targeted to signaling proteins of innate immune responses. Proc. Natl. Acad. Sci. USA.

[171] Hildebrand D., Eberle M.E., Wölfle S.M., Egler F., Sahin D., Sähr A., Bode K.A., Heeg K. (2018). Hsa-miR-99b/let-7e/miR-125a Cluster Regulates Pathogen Recognition Receptor-Stimulated Suppressive Antigen-Presenting Cells. Front. Immunol..

[172] Fleming N.H., Zhong J., da Silva I.P., Vega-Saenz de Miera E., Brady B., Han S.W., Hanniford D., Wang J., Shapiro R.L., Hernando E. (2015). Serum-based miRNAs in the prediction and detection of recurrence in melanoma patients. Cancer.

[173] Shiiyama R., Fukushima S., Jinnin M., Yamashita J., Miyashita A., Nakahara S., Kogi A., Aoi J., Masuguchi S., Inoue Y. (2013). Sensitive detection of melanoma metastasis using circulating microRNA expression profiles. Melanoma Res..

[174] Margue C., Reinsbach S., Philippidou D., Beaume N., Walters C., Schneider J.G., Nashan D., Behrmann I., Kreis S. (2015). Comparison of a healthy miRNome with melanoma patient miRNomes: Are microRNAs suitable serum biomarkers for cancer?. Oncotarget.

[175] Li P., He Q.Y., Luo C.Q., Qian L.Y. (2014). Circulating miR-221 expression level and prognosis of cutaneous malignant melanoma. Med. Sci. Monit..

[176] Kanemaru H., Fukushima S., Yamashita J., Honda N., Oyama R., Kakimoto A., Masuguchi S., Ishihara T., Inoue Y., Jinnin M. (2011). The circulating microRNA-221 level in patients with malignant melanoma as a new tumor marker. J. Dermatol. Sci..

[177] Lawrie C.H., Gal S., Dunlop H.M., Pushkaaran B., Liggins A.P., Pulford K., Banham A.H., Pezzella F., Boultwood J., Wainscoat J.S. (2008). Detection of elevated levels of tumour-associated microRNAs in serum of patients with diffuse large B-cell lymphoma. Br. J. Haematol..

[178] Xin Y., Li Z., Chan M.T., Wu W.K. (2016). Circulating epigenetic biomarkers in melanoma. Tumor Biol..

[179] Solé C., Tramonti D., Schramm M., Goicoechea I., Armesto M., Hernandez L.I., Manterola L., Fernandez-Mercado M., Mujika K., Tuneu A. (2019). The circulating transcriptome as a source of biomarkers for melanoma. Cancers (Basel).

[180] Saldanha G., Potter L., Shendge P., Osborne J., Nicholson S., Yii N., Varma S., Aslam M.I., Elshaw S., Papadogeorgakis E. (2013). Plasma microRNA-21 is associated with tumor burden in cutaneous melanoma. J. Investig. Dermatol..

[181] Sempere L.F., Keto J., Fabbri M. (2017). Exosomal MicroRNAs in Breast Cancer towards Diagnostic and Therapeutic Applications. Cancers (Basel).

[182] Nagesh P.K.B., Chowdhury P., Hatami E., Boya V.K.N., Kashyap V.K., Khan S., Hafeez B.B., Chauhan S.C., Jaggi M., Yallapu M.M. (2018). MiRNA-205 Nanoformulation Sensitizes Prostate Cancer Cells to Chemotherapy. Cancers (Basel).

[183] Osaki M., Okada F., Ochiya T. (2015). MiRNA therapy targeting cancer stem cells: A new paradigm for cancer treatment and prevention of tumor recurrence. Ther. Deliv..

[184] Hosseinahli N., Aghapour M., Duijf P.H.G., Baradaran B. (2018). Treating cancer with microRNA replacement therapy: A literature review. J. Cell Physiol..

